# Positive deviance study to inform a Chagas disease control program in southern Ecuador

**DOI:** 10.1590/0074-02760140472

**Published:** 2015-05

**Authors:** Claudia Nieto-Sanchez, Esteban G Baus, Darwin Guerrero, Mario J Grijalva

**Affiliations:** 1 Biomedical Sciences Department, Tropical Disease Institute, Heritage College of Osteopathic Medicine, Ohio University, Athens, OH, USA; 2 Center for Infectious Disease Research, School of Biological Sciences, Pontifical Catholic University of Ecuador, Quito, Ecuador

**Keywords:** positive deviance, Ecuador, Chagas disease, housing

## Abstract

Chagas disease is caused by Trypanosoma cruzi, which is mainly transmitted by the faeces of triatomine insects that find favourable environments in poorly constructed houses. Previous studies have documented persistent triatomine infestation in houses in the province of Loja in southern Ecuador despite repeated insecticide and educational interventions. We aim to develop a sustainable strategy for the interruption of Chagas disease transmission by promoting living environments that are designed to prevent colonisation of rural houses by triatomines. This study used positive deviance to inform the design of an anti-triatomine prototype house by identifying knowledge, attitudes and practices used by families that have remained triatomine-free (2010-2012). Positive deviants reported practices that included maintenance of structural elements of the house, fumigation of dwellings and animal shelters, sweeping with "insect repellent" plants and relocation of domestic animals away from the house, among others. Participants favoured construction materials that do not drastically differ from those currently used (adobe walls and tile roofs). They also expressed their belief in a clear connection between a clean house and health. The family's economic dynamics affect space use and must be considered in the prototype's design. Overall, the results indicate a positive climate for the introduction of housing improvements as a protective measure against Chagas disease in this region.

Chagas disease affects an estimated eight-nine million people in Latin America, including 230,000 people in Ecuador (PAHO 2006, Hotez et al. 2008 ). Classified as a neglected tropical disease (NTD) by the World Health Organization (WHO), Chagas disease is caused by *Trypanosoma cruzi,* a parasite that can be transmitted through the intake of contaminated food, vertical transmission from mothers to infants during pregnancy or birth and through blood transfusions and organ transplants (Coura 2013). However, the most common mode of transmission occurs through direct contact with the faeces of *T. cruzi *infected triatomines. These insects typically hide during the day in wall cracks, roofs and floors of poorly constructed houses and emerge at night to feed on blood. Symptoms in the acute phase can be similar to those of the common cold and may include fever, fatigue, skin rash, chest pain and enlarged lymph glands. However, the onset of infection can also be asymptomatic. Any symptoms then disappear as most of the parasites are cleared by the immune system and the patient enters the indeterminate phase. Approximately one-third of those infected develop chronic symptoms, five-20 years after the initial infection, as the result of progressive damage caused by remnant trypanosomes that continue to reproduce inside cells and the immune response mounted to combat them. During this chronic phase, Chagas disease can lead to weakness, fatigue and sudden death due to progressive damage and enlargement of vital organs, such as the heart, oesophagus and colon, and destruction of nervous tissue (Crompton et al. 2010).

Despite biological and epidemiological differences, NTDs share characteristics of social and political order that make them prevalent among people living in poverty (Hotez 2008). Among the group of seventeen NTDs, Chagas disease, African trypanosomiasis and leishmaniasis have been particularly neglected in terms of research and drug development (WHO 2012). There is no currently available vaccine for Chagas disease and although medicines such as nifurtimox and benznidazole can be highly effective in the acute phase and are most likely beneficial during the indeterminate and chronic phases (Viotti et al. 2014), both drugs exhibit side effects that become more serious as a patient's age increases. Side effects include allergic dermatitis, peripheral neuropathy and severe hepatic and renal diseases (Bern et al. 2007).

These factors have led to a closer examination of preventive interventions focused on the relationship between Chagas disease and living environments in Central and South America, particularly in rural areas where other factors, such as inefficient health systems, weak infrastructure and limited economic resources, prevent people from frequently assessing their health status. Poor nutrition, weak immune responses and lack of social security systems (Boischio et al. 2009) can also be structurally connected to the high burden of Chagas disease.

The province of Loja in southern Ecuador has showed particular vulnerability to Chagas disease. Our studies in this area indicate a prevalence of *T. cruzi* seropositivity of 3.6% (Black et al. 2007) and triatomine infestation rates of approximately 34.8% in domiciliary units of Loja (Grijalva et al. 2005). Sixteen triatomine species have been identified in Ecuador. Among them, *Rhodnius ecuadoriensis* is the most common. This species has been found in association with sylvatic animals such as squirrels, birds, marsupials and mice, but also with domestic species such as chickens and guinea pigs (Grijalva & Villacis 2009, Grijalva et al. 2012).

Our previous studies have identified other biological and epidemiological facilitating factors for Chagas disease transmission in Loja (Black et al. 2007, Ocana-Mayorga et al. 2010), particularly those related to housing environments that are predominant in this region.

These dwellings are characterised by a predominance of domiciles built with adobe, clay and tile, dirt floors and small windows; there are also peridomiciles where animal nests and food storage areas are attached to the walls of the domiciliary units. The lack of cohesion among walls and roofs in conjunction with the absence of natural light and deep cracks in the adobe walls, as well as accumulation of construction materials, piles of clothes and cluttered environments, facilitate triatomine presence inside domiciliary units (unpublished observations). These structures are also vulnerable to earthquakes, landslides, robbery and increased risk of respiratory infections due to the lack of ventilation systems (Briceño-León et al. 1990).

*Housing improvement as a preventive measure in Chagas disease transmission* - Since Brazilian scientist Carlos Chagas established the presence of triatomines in human environments characterised by "huts with unfinished walls and grass coverage" (Chagas 1909), multiple interventions have focused their efforts on housing improvement as a preventive measure against Chagas disease. Recent studies conducted in Guatemala demonstrated a higher presence of *Triatoma dimidiata* in older houses or houses cleaned less often in rural areas (Bustamante et al. 2009). Similarly, studies conducted in Paraguay (de Arias et al. 1999) showed that the combination of spraying and housing improvement strategies resulted in an efficacy of 100% in vector control interventions. Housing improvement measures applied to protect local houses from *Triatoma infestans* and *T. cruzi*'s presence in this area included smooth, flat and crack-free walls and ceiling surfaces, as well as improvement of ventilation and illumination systems.

Inadequate housing in relation to insect vector control has been defined as the houses' predisposition to pest infestation and inhabitants' illness (Schofield et al. 1990). Accordingly, key aspects to be considered in domestic environments that promote access or multiplication of pests include orientation of the house, vector mode of entry, access to potential food-sources and protective spaces for bug reproduction in domestic and peridomestic environments. Additional elements to be considered by vector control programs that are focused on housing improvement include the availability of materials for construction, population income levels, relationship with local authorities, perception of access to alternative models of housing, possibilities of collaboration between designers and local inhabitants and land tenure policies (Cairncross et al. 1990).

*Participatory decision-making in health promotion initiatives* - Several policy documents prepared by the WHO, Pan-American Health Organization and the Centers for Disease Control and Prevention, among others, have stated the relevance of fostering community involvement in health interventions, including the Alma Ata Declaration (1978), Ottawa Charter (1986), One World, One Health Declaration (2004) and Rio Political Declaration on Social Determinants of Health (2011). All of these organisations recognise the interdependence of environmental, political and social factors in the preservation of human health and the need to develop decision-making processes that are conducive to facilitating individual and community participation.

Consequently, recent health interventions have experienced a conceptual and practical shift from theories oriented to individual behaviour change toward socio-ecological perspectives aimed at encouraging the participation of people who are directly affected by health issues in coordination with local, national and international organisations (Obregon & Mosquera 2005). This approach does not dismiss the relevance of behaviour change, but considers it as a possible result of a process that is mainly focused on fostering the "capacity of community members to actively participate in defining the scope of health problems that are relevant to them and determining corresponding and equally relevant solutions" (Airhihenbuwa & Dutta 2012). Perspectives such as global health communication (Obregon & Waisbord 2012) and the culture-centred approach to health communication (Dutta 2008) conceptualise health promotion interventions as processes through which people understand their health priorities under specific social contexts through participatory decision-making. Participatory decision-making is deemed particularly effective to mobilise networks and leaders, create political will, increase knowledge, change attitudes, ensure individual and community demand for services and reach out to marginalised populations (Obregon et al. 2009).

*Healthy Homes for Healthy Living (HHHL) project* - We have determined that traditional Chagas disease control strategies employed in Ecuador, such as active searches for triatomines, insecticide-based fumigation and community education activities, are effective at reducing triatomine infestation in the short term, but do not prevent re-infestation in the long run (Grijalva et al. 2012). As evidenced by studies conducted in Loja and Manabí, triatomines rapidly re-colonise housing units once the insecticide residual effect subsides. Consequently, we formulated the Healthy Living Initiative (HLI), a comprehensive strategy aimed at understanding and promoting the improvement of the socioeconomic conditions of rural communities affected by Chagas disease in southern Ecuador. To enable access to better living environments, the HLI has facilitated projects such as the construction of drinking water systems, formalisation of income-generation initiatives and creation of collaborative efforts aimed at strengthening negotiating skills among local leaders and regional authorities in three communities in southern Ecuador. Community members have also received information and participated in educational activities about Chagas disease *via* family, school and community-based initiatives. In this context, the HLI proposes the HHHL project, a health promotion strategy aimed at interrupting Chagas disease transmission by creating living environments designed to prevent triatomines from colonising houses. These living environments consider the infrastructural conditions of the house and peridomestic areas as well as the practices associated with hygiene and the organisation of the space according to the cultural and economic activities of the region. Given the limited effectiveness of traditional control strategies, the HHHL proposes home improvement as a basic condition to sustainably prevent Chagas disease and holistically promote health at the household level. This proposal is consistent with existing public policies being implemented in Ecuador during the last decade as part of the Good Living National Plan (SENPLADES 2015).

This study used the positive deviance (PD) methodological approach to conduct formative research aimed at informing decision-making in the design of an HHHL housing prototype adapted to this region's social, geographical and cultural characteristics. As an asset-based theoretical and methodological approach to community development, PD seeks to identify resources that are already used by local populations to solve challenges they face on a daily basis. The premise is that PD-led interventions can be highly effective "because the community owns the solution, self-discovers it through dialogic inquiry and there is 'social proof' that those ideas can be implemented locally with no extra resources" (Singhal 2011). We sought positive deviants - "individuals whose uncommon practices and behaviours enable them to develop better solutions to problems than their neighbours who have access to the same resources" (Singhal et al. 2010) - to identify knowledge and practices of local families concerning the physical structure, maintenance, hygiene and organisation of houses that are deemed to be protective against the presence of insects. In addition, this research was aimed at establishing local attitudes toward the idea of a "healthy home" as a protective measure facing Chagas disease.

## MATERIALS AND METHODS

*Study area* - This study was conducted in the communities of Chaquizhca, Bellamaria and Guara, located in Calvas County, Loja. Loja has a dry, temperate climate and vegetation that is primarily dominated by bushes (Grijalva et al. 2012). Precarious socio-economic conditions of the families in these communities are brought on by limited job opportunities, limited access to resources such as clean water and sanitation in their homes, as well as limited access to health providers, medicines and health education. Most families work small plots of land that provide them with enough food to feed their families, but not enough to facilitate an important participation in local markets (unpublished data).

*Selection criteria* - Participants were purposely selected using two criteria. First, the absence of triatomines in domiciliary units in 2010-2012 for Chaquizhca and Guara and 2011-2012 for Bellamaria. Entomological data collected by our group in collaboration with the National Chagas Control program in 2010, 2011 and 2012 was reviewed, showing that 53 (40.8%) of the 130 houses located in the study area have shown triatomine presence at least once (unpublished data). Participants were selected from the additional 59.2%. Second, the predominant use of traditional construction materials (adobe walls, tile roofs and dirt floors) in their domiciliary units. Pictographic decay analyses and blueprints of each one of the houses also collected by our group in 2012 were used to secure a selection of participants who share similar characteristics (Pascale et al. 2010) in relation to housing structures. Thirty-seven houses out of the 130 existing in the communities followed the previously described criteria and 11 of them were finally selected based on similarities in terms of the number of inhabitants, distance to the main road, longevity of the construction and land tenure. Tenants whose houses have registered triatomine presence in the same periods of time or were built with non-traditional materials were excluded from this study.

*Study design* - Interviews, photos and participatory sketches were used to elicit existing knowledge, attitudes and practices related to the construction, organisation, cleaning and hygiene of insect-free houses.

*In-depth interviews* - A semi-structured interview protocol was designed to explore the knowledge and practices related to the physical construction and the use of the space in intra and peridomiciles, as well as attitudes toward the idea of a "healthy home"*. *Participants from triatomine-free houses were asked about construction, organisation and cleaning practices. The interview protocol was validated by HLI local personnel to adjust it to local language prior to its application. Interviews lasted from 30-60 min and were personally administered by the researcher supported by a vernacular translator.

*Photographs* - To recognise the values and meanings involved in the concept of "home", participants were asked to take one or two pictures of their favourite spot in their houses and to describe the reasons for this selection. Some participants expressed a preference for the researcher to take these pictures under their guidance. Narratives explaining the reasons for this selection were guided by the photo-voice SHOWeD method (Wang 2006) and translated into daily routines by a vernacular translator.

*Focus groups* - A focus group was conducted to validate practices identified by positive deviants during the in-depth interview phase. The focus group included three stages: (i) Photo-elicitation - Photo-elicitation is a method used in visual anthropology to introduce pictures into research interviews (Lindlof & Taylor 2011). Pictures taken during the interview phase were presented to focus group participants. Using these pictures as reference, they were asked to describe their idea of a "healthy home". The generative question used is this exercise was *"*Can you see a healthy practice in this picture?*"*. (ii) Participatory sketching - Participatory sketching is a method of collective drawing employed to obtain enriched narratives from research participants. It has been used in previous PD studies (Greiner et al. 2007). Participants were divided into subgroups to jointly draw a sketch describing what they envision as a healthy environment at home in intra and peridomestic areas. Sketches were then presented to the entire group. (iii) Discussion - Participants were encouraged to use pictures and drawings to discuss housekeeping and organisation practices at home that could lead to a healthy environment in their communities. Finally, they were asked to identify which of the previously discussed practices could be immediately implemented in their own homes and which ones other community members could easily replicate.

*Data analyses* - The information obtained from interviews and focus groups was transcribed in its entirety. A thematic analysis (Creswell 2009) was conducted and all the information was coded according to four categories: house construction techniques, practices and the use of the space in intradomiciles, practices and the use of the space in peridomiciles and attitudes toward home improvement. Pictures and drawings were similarly coded. Because the information was collected in Spanish, the writing process required a translation stage that was limited to the most relevant information in each categorical code. An ethnographic approach (Foley & Valenzuela 2005) was applied to capture the multiple dimensions of participant lives in their cultural, social, political and economic complexities.

*Ethics* - This study design was approved by Ohio University's Institutional Review Board (protocol 12X113). Interview participants were individually visited in their homes, introduced to the research's goals and invited to be part of this study. Informed consent forms were signed and collected from all participants, including permission to show pictures taken during the interviews to other community members and stakeholders.

## RESULTS

Eleven interviews were conducted with the participation of the heads of households that had not shown triatomine infestation over time (positive deviants). A focus group with six community members was also conducted to validate the practices described by positive deviants. The results derived from their responses were organised according to the categories derived from the previously mentioned thematic analysis.

*Houses' construction techniques* - "The walls of my house are made out of adobe covered with cement and sand to reduce the weathering caused by animals and even by us. We built this house 45 or 50 years ago [...] We used lots of cement and we've never had to repair this plastering. The roof is made out of clay tiles now; it was initially made out of cane and hay. The floor is all dirt because dirt is better than cement: cement is too cold and as we are old, it can cause bone pain."

Most of the houses located in these communities follow the same construction parameters described by this participant, except for the wall plastering. Compared to bahareque structures built with mud and bamboo by previous generations, adobe houses represent an improvement in terms of safety and social status. Although all participants indicated that a basic structure of adobe walls, tile roof and dirt floor was chosen because it was all that they could afford when they built their houses, they also provided an extensive set of reasons to maintain this model throughout the years.

One participant expressed that adobe constructions are more comfortable in terms of the temperature inside the house compared to the model provided by the national government through the Ministry of Urban Development and Housing (MIDUVI). "What makes the MIDUVI houses so hot inside is the ardex roof (corrugated sheets made out of cement fibre and polypropylene strips). Bricks and cement walls are fine, but adobe walls are cooler." This statement coincides with previous studies conducted by architects from the Catholic University of Ecuador who visited this region in July 2012 and established thermal differences of 5-10ºC between MIDUVI and traditional houses.

Traditional knowledge is another reason for choosing this type of construction. Participants expressed that it is very common for families in this region to be directly involved in the actual construction of their houses and only pay for labour when they need help or specialised knowledge. Different from cement or bricks, local populations know how to prepare the adobe with water, mud, hay and cow or donkey manure. They do not have to pay for the raw materials as it is naturally available in their communities. Similarly, tiles are more convenient than other roofing techniques such as zinc sheets because if one breaks, they can easily replace it at an affordable cost. They can also collect *carrizo* (*Arundo donax*) and wood to replace deteriorated sections of the roof if needed.

**Fig. 1:**
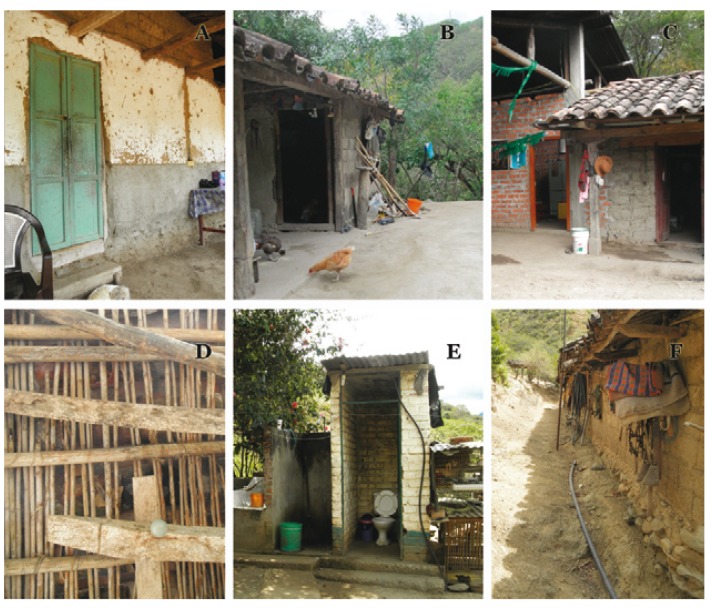
positive deviants have implemented different solutions to deter the natural decay of their houses and reorganise their living environments. Improvements applied to traditional adobe structures include: cement plastering (A), cement floors in the porch area (B), partial additions constructed with bricks (C), carrizo ceiling and chahuarquero beams (D), latrines and water tanks (E) and rock foundations (F).

Even though some participants think adobe houses are more beautiful and safe than bahareque constructions, others have chosen bricks and cement as alternatives to reinforce or extend their basic constructions. Similarly, some participants have explored alternatives to solve the natural decay of their houses. Chonta palm (*Bactris gasipaes*, also known as *pambil*) has been extensively used as a long-term solution to reinforce roofing structures. Although this material is not naturally available in the region and has to be bought in the local market, it provides advantages such as long-lasting protection against plagues such as moths. Due to its natural dimensions (approximately 3 m long and 7 10 cm wide), chonta wood is commonly used as support beams and columns to build roofs. Similarly, *Chahuarquero*, the 3-4 m long thin stem of the *cabuya* plant (*Furcraea andina*), in combination with bamboo is often used as a temporary solution to support clay tiles when there is extensive damage. *Cha-* *huarquero*, being a low quality wood, is a short-term solution, but has the advantage of being naturally available and present in most of the plots where local farmers grow their products. Other variations of traditional construction employed by positive deviants include rock barriers to strengthen fences and foundations, a mix of bricks and adobe in certain areas of the house, *carrizo* ceilings and cement floors in porches and rooms (Fig. 1).

Traditional forms of funding through family loans and animal commerce can impact house construction time. One of the participants identified this situation as problematic because it is a result of the limited resources available to them on a regular basis. Two years ago, he built the walls of a new room for his house with an elevated zinc roof that leaves a considerable gap between the roof and wall. He had not finished the construction because he was trying to save the money necessary to complete it. In the meanwhile, his daughter and grandchildren had occupied this area, with an increased exposure to rain, bugs, mosquitoes and other environmental factors. "If things are not finished it's not because we're careless, but because we do not have enough resources. We all work very hard here: myself, my wife, my sons and daughters, even the ones who live far from us help."

*Practices and the use of the space in intradomiciles* - After his 18-year-old son died from leukaemia, one of the participants took several measures to protect his family's health, the most important one was to fumigate every time he sees any scorpion, spider, or flea. "The most important thing is to be clean (...) People have to be careful, prevent and be watchful of their own health. I always tell my friends that they have to clean, remove, fumigate and have their house as clean as possible. We kill the bugs when we see them and by killing the bugs we also kill that Chagas parasite." Some of the substances used in this practice were originally formulated to combat cattle plagues, including commercial products that contain different formulations of malathion, dichlorvos and carbaryl. Whether regular or occasional, all the participants referred to self-fumigation as the most effective preventive measure to control bug presence in intradomiciles.

Positive deviants consistently referred to the concept of hygiene as a priority at home. Its relevance as a social norm is illustrated by expressions such as "cleanliness is a virtue", "it doesn't matter how poor you are, you have to clean your house" and "cleanliness is an individual matter (...) I don't care how old or poor my house is: I sweep it every day!". However, the local weather and natural environment create persistent circulation of dust and sand, particularly during the dry season. Consequently, local families have taken measures to keep their houses as clean as possible under these circumstances. The most important one is to keep windows and doors closed most of the time, especially when they are not at home. Windows are normally small and covered with wood. Although the circulation of air and natural light is reduced as a result of this practice, it constitutes a protective barrier in the absence of glass, screens or other protective measures. Participants also mentioned safety issues related to theft as a reason to keep doors and windows closed most of the time.

**Fig. 2:**
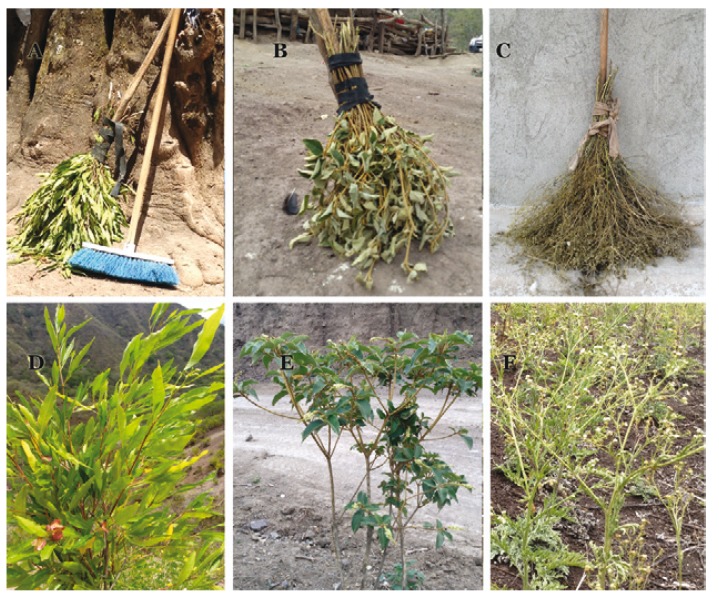
species of bushes believed to have natural insecticide properties and used as brooms by positive deviants. A-D: chamana (Dodonaea viscosa); B-E: moshquera (Croton sp.); C-F: florblanca or monteramirez (Buddleja utilis).

Some positive deviants also reported practices such as sweeping more than once a day in intra and peridomiciles, using water to help dirt stick on the floor and create less dust and sweeping with brooms made from plants. Bushes such as *porotillo* (*Fallopia convolvulus*), *moshquera* (*Croton *sp*.*), *florblanca* (*Buddleja utilis, *also known as *monteramirez*) and *chamana* (*Dodonaea viscosa*) are believed to be highly acidic plants by local populations; when turned into brooms, they become a natural insecticide (Fig. 2). These bushes are also used to sweep walls and the interior corners of roofs: "My husband and son sweep the walls up to the roof at least once a month to avoid little spiders and spider webs. We clean with *florblanca* or *monteramirez* and you can see that in my house there is not even one flea.". Participants also reported exposing blankets and bedclothes to sunlight on regular basis.

Finally, one of the positive deviants explained that it is common to use *soberados* to store crops and seeds in these communities. *Soberados* are false ceilings built with *chahuarquero* located in the kitchen with the purpose of using the smoke produced by firewood stoves to protect crops from moths. Except in this case, all the houses lack spaces specifically designed to store food and clothes. As a result, local families keep their clothes in nylon sacks, plastic cans or cardboard boxes. Hanging them from nails and strings attached to walls and roofs is also common.

*Practices and use of the space in peridomiciles* - Our previous studies established that *R. ecuadoriensis* and *Pastrongylus chinai* are commonly found in peridomestic habitats in association with chicken coops (Grijalva et al. 2005), guinea pig pens, wood piles and rat nests (unpublished observations). In spite of this entomological risk, animals are closely tied to local traditions and economic activities in these southern Ecuador rural communities. Guinea pigs, for example, are considered not only a delicacy for special occasions, but also an important source of income. In addition to their cultural and economic value, traditional knowledge describes guinea pigs as sensitive animals that could die if exposed to unstable environmental conditions. Therefore, it is believed that they have to be kept in kitchens or rooms where people can watch them and provide some sort of heat that helps them to grow better. However, none of the participants keep guinea pigs in their rooms and only two of them reported keeping them in the kitchen.

Most of the participants have relocated animal coops, crops and residual materials from areas attached to the houses' walls and ceilings to new spaces in the peridomicile. Traditional chicken coops made out of banana leaves known as *cushas*, have been replaced by alternative structures made of *chahuarquero*, planks, rags, pieces of bricks and paperboard. Another participant reported using a papaya tree close to his house to keep the chickens far away from the walls. These actions are combined with other practices such as fumigation with insecticides when nests are assembled and nest burning after chicks hatch (every 22 days). This fumigation is specifically applied to protect nests from *cundil* or *uruzungo* (*Menacanthus *sp*.*). Other animals such as dogs and cats are kept around during the day but they are not allowed to sleep inside the house.

Other practices are focused on residual materials and waste management. Although some of the participants have gas stoves, most of them continue to use their wood stoves; for that reason, they need to keep firewood around the house. Rustic covered structures made out of wood sticks and basic roofs have been specifically created with the purpose of protecting firewood from rain. Other practices among positive deviants include cleaning and chopping wood sticks before piling them. As firewood is chopped, it occupies less space and can be more effectively stored. Participants reported burning waste every three-five days. Plastic materials are usually stored in nylon bags until there is enough to burn, while organic residuals are spread and employed as natural fertiliser in orchards and plots.

Another common element identified in peridomiciles of positive deviant households is sanitary services built with support from institutions such as the Emergent Social Investment Fund, Uriel Foundation and the local government (Autonomous Decentralised Government of the Canton Calvas) at different times during the last 10 years. Although these structures are present in most of the households, they are not always functional and in some cases, have been adapted to serve as storage or animal shelters. Some reasons argued in this respect include a lack of maintenance, missing or broken pieces or even incomplete initial construction.

*Attitudes toward home improvements* - All the interviewees expressed interest in improving their houses and a willingness to invest labour and personal resources in this endeavour. Some of them recognised imminent risks derived from the thinness of the walls or steep slopes affecting their homes' structure, while reporting that they have no money to pay for what is needed to repair them. Participants stated that major improvements to their houses would not be possible if they do not have access to some external support. The research participants would like to implement several improvements in the near future: bigger windows covered with glass, replacing roofs with safer and more durable materials, higher elevation of ceilings and walls, wall plastering, installation of *chahuarquero *ceilings and construction of additional rooms. Comments about cement floors were divided among those who would like to have them to facilitate daily cleaning and those who consider them cold and potential sources of rheumatic and respiratory diseases.

The Table provides a summary of the practices and knowledge shared by research participants about their homes and the protective measures they have taken to prevent their natural deterioration. Knowledge and practices identified during the interviews were shared in a focus group where community members validated their effectiveness as preventive measures against the presence of bugs. Pictures and stories collected during the individual interviews were used to spark dialogue during the focus group. These images lead participants to voice their attitudes toward home improvements using their own definitions of health: "Health is having the house clean and healthy for people's lives." "Health is to have access to basic services." "Being healthy is to have good conditions of life and good hygiene." "It is to have the house very clean and fumigate often to kill bugs." "Health is the most important thing. One can have the best of life, but if one is sick, it does not matter." "Being healthy is to be active. One has to make an effort to work even if one is sick.".

**TABLE:** Practices and knowledge reported by heads of household that have remained ttriatomine free in entomological searches conducted in 2010, 2011 and 2012

Practices	Knowledge
House structure	
Traditional houses are built with adobe walls, dirt floor and tile roofing.	Adobe constructions are cooler than Ministry of Urban Development and Housing houses.
All the families have carried out some sort of improvement in their houses in the last 10 years.	People in the region know how to build and repair traditional constructions.
Construction can take a long time until completion.	Bricks and cement constructions can reduce dust presence inside the houses.
Selling livestock, working for somebody else or using the solidarity bonus (government assistance) are some of the income sources used by local families to fund house improvement projects.	Families are normally directly involved in building their houses.
Intradomiciles	
Chemical fumigation is referred to as the most important protective measure against triatomines**and other plagues. Fumigation is applied in the domicile and peridomicile.	Hygiene is highly valued as a social norm in this region.
Animals are not allowed to sleep inside the house.	Plants used as brooms include *porotillo *(*Fallopia convolvulus*), *moshquera* (*Croton* sp.), *florblanca* (also known as *monteramirez *(*Buddleja utilis*) and *chamana* (*Dodonaea viscosa*).
Windows are kept closed to reduce dust circulation inside the houses.	Having *soberados* (false ceilings) on kitchen roofs helps to protect crops from moths and serves as storage space.
People usually sweep more than once a day in the domicile and peridomicile. It is common to use water to help dirt stick to the floor and create less dust.	Local people use a mix of western-style health care services, traditional healers and medicinal herbs to take care of their health.
Brooms are made out of local plants believed to be natural insecticides or to have an insect repellent effect. They are used to sweep walls and roofs.	
Changing bedding and exposing blankets to sunlight is also considered a protective practice.	
Peridomiciles	
Positive deviants have moved chicken nests to trees and corrals relatively far from their houses’ walls.	The relationship between animals and local populations is closely tied to traditions and economic activities.
Traditional chicken coops made out of banana leaves or other organic materials known as* cushas *(chicken coops) have been replaced by alternative structures made out of materials such as bricks, wire mesh, *chabuarquero *or *cabuya* steam (*Furcrcea andina*), planks and rags.	Guinea pigs have to be kept in kitchens or rooms where people can watch them and provide some sort of heat that help them to grow better.
Chicken coops are also fumigated when assembled and burned after chicks hatch.	Wet firewood is useless. People protect firewood from rain by creating basic coverage structures, chopping and cleaning it on regular basis.
Organic waste is used as natural fertilizers.	Porches are subject to public scrutiny and are kept as clean as possible

Open-air circulation was usually mentioned when addressing desirable features of a healthy home. Similarly, a nice view is highly valued as a way to be in touch with other families and the natural environment. Porches excel as social spaces and are used as dining rooms, work spaces and social areas. Participants expressed that this space is subject to public scrutiny and should be kept as clean as possible. In the words of one participant, "it is important to sweep outside every day because we have many friends. The house has to look clean in case they want to come to visit. That's why we clean all around the house every day".

**Fig. 3:**
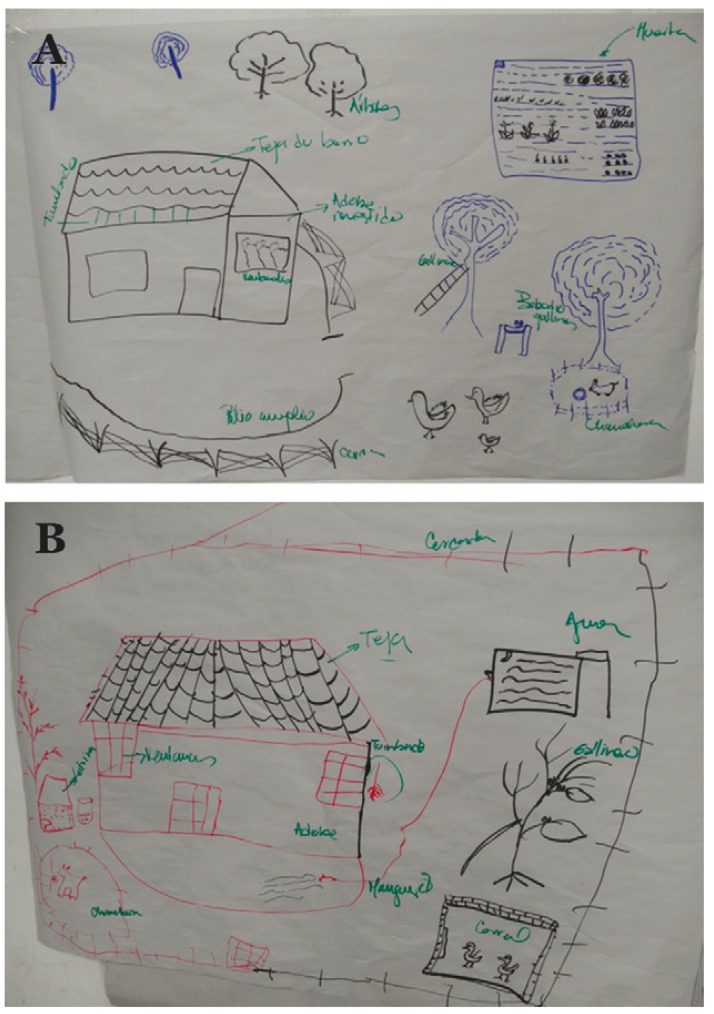
elements of a healthy house described in the focus group using participatory drawing. A: plastered walls, big glass windows, backyard, fence, pig breeding, orchard for medicinal plants and vegetables and chicken coop; B: garbage can, pig breeding, latrine, backyard hose, plants, water tank, tumbado (ceiling), tile roof, chicken coops by the trees, fence, entrance door, windows and adobe walls.

Elements of a healthy home mentioned in the participatory drawing exercises (Fig. 3) included garbage cans, pig corrals, latrines, backyard hoses, gardens for aromatic plants, water tanks, ceilings, tile roofs, bigger windows, orchards, adobe walls, chicken coops and fencing around the peridomicile featuring an entrance door to prevent entry by domestic and wild animals.

When asked to describe why the houses included in the drawings were healthy, participants stated: "These houses are healthy because the animals are outside." "Plants are part of the environment. We need them to breathe fresh air. The trees are also good because they provide shade for the animals; otherwise they are going to look for protection from the sun inside the house." "There is water in the house and that helps us to clean up often." "It is good to have medicinal plants because sometimes you get sick and you just need an *agüita* (infusion) to feel better. Growing organic onions and lettuce, with no chemical substances, is also good for your health". "It is a beautiful house. You feel like you would like to live there.".

In accordance with the PD methodological approach, practices such as relocation of chicken nests, the use of natural insecticide brooms and regular sweeping of walls and roofs were identified by focus group participants as practices that are easily actionable and replicable by other families in these communities.

## DISCUSSION

This research provided important information on the practices and knowledge regarded as protective against triatomine presence by the families of communities involved in this PD research. Although none of the practices listed can be considered individually sufficient to achieve vector control, they constitute the body of knowledge displayed by research participants when involved in reflections focused on their living environments.

Drawings and pictures derived from this research provided initial ideas for the design of a healthy home prototype adapted to the geographical, cultural and social characteristics of these communities. The healthy homes models favoured by research participants do not drastically differ from the currently existing houses in the communities, suggesting that elements of the local living environments can be improved in order to interrupt Chagas disease domiciliary transmission cycle while preserving local ways of living. Moreover, local communities expressed associations between living environments and health, suggesting a positive climate for the introduction of the idea of housing improvement as a protective measure against Chagas disease in this region.

Our results indicate there is a base of local knowledge that should be considered to create sustainable control of Chagas disease transmission in this region. Adobe constructions, for example, were regarded by research participants as socially acceptable, affordable and useful housing solutions despite the entomological risk they could represent. Although it is widely known that cracks and gaps in walls and ceilings can result from using adobe as the main construction material (Enger et al. 2004, Black et al. 2007, Mischler et al. 2012, Saunders et al. 2012), all the interviewees used it in their own homes along with locally created maintenance techniques such as plastering and regular cleaning of walls and roofs. Therefore, adobe-based construction techniques can be considered for the construction of an anti-triatomine house model if current adobe-making techniques are improved or plastering solutions are incorporated. Other local materials such as *carrizo* and *chonta* can also be considered as they are known to be durable and desirable in terms of housing. Cement floors can generate certain resistance due to their association with respiratory and rheumatic diseases. Alternative solutions such as a stabilised mud cement floor should be considered. Additionally, involving the local population in the actual construction of the house could be an effective option to facilitate community ownership of the HHHL project and reduce the labour costs associated with infrastructure improvement.

This PD study also provided information about the productive and social practices within the home, which is essential for our holistic understanding of the Chagas disease transmission dynamics within the context in which it develops rather than as an isolated phenomenon. For instance, interactions between families and domestic animals are central to the rural environment of the communities included in this study. None of the research participants reported having domestic animals living inside their bedrooms. Instead, most of them have created alternative structures to raise chickens and guinea pigs in the peridomestic area. It is important to consider, however, that local families wish to be close to their animals due to their value as sources of food and income, in addition to their uses as pets and home protection. Alternative structures for domestic animals should consider their cultural and economic value.

Similarly, specific structures should be designed to support other economic activities conducted in and around the domestic unit, such as crop drying and storage, as well as traditional jam and raw sugar making. During the interviews, we observed sacks with freshly picked products piled up in kitchens, rooms and halls around the house. One participant replied to the questionnaire while selecting coffee grains usually stored in the living room of his house and most of them husked corn and threw it to the chickens during our visit. Providing spaces for proper storage of clothes, agriculture products and seeds should be considered in the design of the HHHL prototype.

Other economic activities such as goat grazing, cattle raising and agriculture can lead to soil erosion, with the natural loss of humidity responsible for dust circulation in this environment. Further research should be conducted to understand how the practices used in this region contribute to the risk for domiciliary triatomine re-infestation. Similarly, occupation of unfinished constructions due to the absence of sufficient resources to complete them can increase the local population's exposure to Chagas disease vectors.

Finally, according to research participants, chemical fumigation is an extended preventive measure against plagues in this region. Although the effects of self-application of different agricultural and domestic-grade insecticides on human health are not clear, these products are commonly applied in the domicile and peridomicile, as well as in chicken coops and guinea pig pens. Further research should be conducted to determine the effects of substances that are currently used by local communities on human health. Similarly, our results suggest that chemical insecticide spraying constitutes a fragile short-term solution for Chagas disease control because multiple applications are needed to control bug re-colonisation, particularly in poorly constructed houses. This indicates the need to develop long-lasting and sustainable solutions for Chagas disease transmission in areas that are at high risk of triatomine infestation.

There are important limitations in our study that must be addressed to contextualise our results. First, the fact that the houses selected showed a consistent absence of triatomines during entomological searches conducted in collaboration with the National Chagas Control Program does not necessarily imply that these houses are bug-free throughout the year. In fact, all the entomological searches were conducted in the summer season; therefore, natural factors associated with the presence or absence of triatomines in rural dwellings in other times of the year remain unknown. Similarly, it is likely that the practices reported by positive deviants are not exclusive of this group of individuals and that different combinations of these practices as well as consistency in their application increase the possibility of positive deviants obtaining better results than their neighbours. However, if families whose houses have registered triatomine presence are already implementing some of these practices, this limitation can easily turn into an asset by focusing health promotion interventions on making local families aware of their advantages for protecting their families' health.

Another limitation derived from the participatory methods implemented in this research was the discomfort expressed by some participants when they were asked to use photo cameras and drawings. This was addressed by the participation of a local field technician with permanent presence in these communities who turned research questions into the language and activities that are familiar to local populations and played the role of a vernacular translator. His knowledge and ongoing relationships with local communities was instrumental in facilitating the dialogue between internal and external actors.

Poverty was referred to as the most decisive element in choosing these traditional techniques for housing construction. Participants reported that adobe is substantially more affordable than other construction materials because they know how to make it with naturally available resources, which represents an important socio-economic advantage as it eliminates the need to pay for labour and raw materials. This is a crucial element in the context of Chagas as a disease of poverty. The WHO introduced the category of NTDs or diseases of poverty in 2005 as a result of a paradigm shift within the organisation that made the impact of tropical diseases on populations living in poverty a global priority (Smith & Taylor 2013). Consequently, the WHO has recommended particular attention to the political, economic, social and cultural needs expressed by populations affected by NTDs to create effective interventions aimed at delivering integral treatment and strengthening local health systems.

Exploring the potential of participatory health promotion processes to break the cycle of neglect that defines NTDs remains an important endeavour that could facilitate productive dialogue between the people affected by diseases, decision-makers, donors, policy designers and researchers (Ventura-Garcia et al. 2013). By recreating the stories shared through the dialogical interactions that occurred during this PD research, we aimed to approach the research participants and communities beyond labels such as neglected, poor, at risk, sick or isolated, to be able to share complex ideas about the local knowledge, expertise, culture and expectations, all of them associated with the idea of a healthy living environment.

Securing access to decision-making instances along the process of definition, implementation and evaluation of health interventions can provide important insights to facilitate meaningful participation of populations that are at risk for NTDs. Although the construction of the HHHL prototype constitutes an external solution, its effectiveness will largely depend on the use and appropriation that local families make of the new space. Our results suggest that community members are enthusiastic about sharing their knowledge and are willing to test house improvement solutions. This PD research provided valuable data to frame health promotion strategies associated with the introduction of the HHHL project with context-specific information derived from knowledge and practices currently applied by community members. It also provided important information to adapt the design of the HHHL prototype to economic, cultural and social dynamics structurally associated with Chagas disease as a disease of poverty. Most importantly, this PD research illustrated an environment of positive collaboration that can potentiate community engagement in the construction of healthy communities and other goals beyond the scope of HHHL. Invitational approaches to health promotion such as dialogic social change (Greiner et al. 2010) can be used to encourage agency and autonomous engagement of local populations and provide a wider definition of community participation in decision-making processes derived from complex analyses of disease dynamics from a socio-ecological perspective.

In this context, it is important to consider the political implications of house improvement as a long-term control strategy for Chagas disease. Local and national governments, as well as local communities, must be engaged in the generation of viable solutions that could be jointly founded and implemented. The formulation of effective and comprehensive solutions requires a dialog aimed at preventing institutional silos within the public and private sectors. Therefore, a multidisciplinary approach is necessary to articulate health, infrastructure, income generation and social organisation processes necessary to guarantee effectiveness and affordability of protective measures in agreement with local livelihoods. Consequently, an extended conversation is needed in terms of policy making at the level of the Ministry of Health of Ecuador and the National Chagas Control Program and their counterparts at the Ministries of Housing, Development and Social Inclusion, Education and Agriculture. Non-governmental organisations and at risk communities should also be included to consider their appreciations about the benefits and limitations of house improvement as long-term disease control measure. The current Ecuadorian administration formulated the National Development Plan for Good Living 2013-2017 (SENPLADES 2013), which seeks such an approach. Therefore, there is a political discourse and environment currently in place in the country that could fulfil the goals of social and territorial equity, cohesion and inclusion, while effectively protecting vulnerable populations from Chagas disease.
